# Lead location as a determinant of motor benefit in subthalamic nucleus deep brain stimulation for Parkinson’s disease

**DOI:** 10.3389/fnins.2022.1010253

**Published:** 2022-10-04

**Authors:** Jerrold L. Vitek, Rémi Patriat, Lisa Ingham, Martin M. Reich, Jens Volkmann, Noam Harel

**Affiliations:** ^1^Department of Neurology, University of Minnesota, Minneapolis, MN, United States; ^2^Department of Radiology, Center for Magnetic Resonance Research, University of Minnesota, Minneapolis, MN, United States; ^3^Consultant, Minneapolis, MN, United States; ^4^Department of Neurology, University Hospital Würzburg, Würzburg, Germany

**Keywords:** Parkinson’s disease, deep brain stimulation, subthalamic nucleus, brain imaging, lead localization

## Abstract

**Background:**

Subthalamic nucleus (STN) deep brain stimulation (DBS) is regarded as an effective treatment for patients with advanced Parkinson’s disease (PD). Clinical benefit, however, varies significantly across patients. Lead location has been hypothesized to play a critical role in determining motor outcome and may account for much of the observed variability reported among patients.

**Objective:**

To retrospectively evaluate the relationship of lead location to motor outcomes in patients who had been implanted previously at another center by employing a novel visualization technology that more precisely determines the location of the DBS lead and its contacts with respect to each patient’s individually defined STN.

**Methods:**

Anatomical models were generated using novel imaging in 40 PD patients who had undergone bilateral STN DBS (80 electrodes) at another center. Patient-specific models of each STN were evaluated to determine DBS electrode contact locations with respect to anterior to posterior and medial to lateral regions of the individualized STNs and compared to the change in the contralateral hemi-body Unified Parkinson’s Disease Rating Scale Part III (UPDRS-III) motor score.

**Results:**

The greatest improvement in hemi-body motor function was found when active contacts were located within the posterolateral portion of the STN (71.5%). Motor benefit was 52 and 36% for central and anterior segments, respectively. Active contacts within the posterolateral portion also demonstrated the greatest reduction in levodopa dosage (77%).

**Conclusion:**

The degree of motor benefit was dependent on the location of the stimulating contact within the STN. Although other factors may play a role, we provide further evidence in support of the hypothesis that lead location is a critical factor in determining clinical outcomes in STN DBS.

## Introduction

Deep brain stimulation (DBS) of the subthalamic nucleus (STN) is an effective treatment for reducing motor symptoms and improving quality of life in patients with advanced Parkinson’s disease (PD). While it is generally accepted that STN DBS can improve motor function in PD, the degree of benefit can vary significantly across centers and within centers across patients (Kleiner-Fisman et al., 2006; Zaidel et al., 2010; Wodarg et al., 2012). Indeed, in some patients undergoing STN DBS motor signs show no improvement or may worsen (Okun et al., 2005; Ellis et al., 2008; Tripoliti et al., 2011; Wodarg et al., 2012; Rossi et al., 2018).

Among those performing STN DBS many agree the sensorimotor region is the optimal target within the STN for implantation of the DBS electrode, though others debate this (McClelland et al., 2009; Caire et al., 2013; Kasasbeh et al., 2013). Some have reported that variations in lead location do not correlate to clinical outcome (McClelland et al., 2009; Caire et al., 2013; Kasasbeh et al., 2013), while others report a significant difference in lead locations with dorsolateral implants resulting in superior outcomes (Wodarg et al., 2012; Garcia-Garcia and Guridi, 2016; Horn et al., 2017a).

Attempts to define the best location for stimulation within the STN has many challenges. Bias in the methods for assessing motor signs, whether patients are in a practically defined off medication state or are fully optimized with DBS, differences in phenotype, and the ability to accurately determine the location of the lead with respect to the anatomical structure are all variables that may confound our interpretation of the role of lead location in determining clinical outcome (Post et al., 2005; Evers et al., 2019).

The lack of patient-specific imaging technology that can accurately identify the precise location of the lead and its contacts for each patient is likely a critical factor in the disagreement over the optimal stimulation site within the STN. While several imaging approaches have been used, they are generally based on variations of a singular atlas for mapping anatomical locations rather than each patient’s STN (Nowacki et al., 2018; Ewert et al., 2019). Given the demonstrated variability between patients in the size, shape and geometric configuration of the STN, atlas-based methods for determining lead location can be fraught with inaccuracies because they do not consider the anatomic variability that exists across patients (Duchin et al., 2018).

The implanted location of DBS electrodes is very likely a critical predictor of clinical outcome (Welter et al., 2014; Bot et al., 2018; Schrock et al., 2021). This study is intended to evaluate whether using new visualization technology with precision of less than one millimeter is able to demonstrate a clear and significant relationship of lead location within specific regions of the STN to motor outcomes.

## Materials and methods

### Subjects

This is a retrospective study that included 44 patients from a previously studied cohort implanted at Würzburg University Hospital in accordance with the Declaration of Helsinki and approval of the internal review board. These patients previously formed a cohort to examine the role of anatomic and functional connectivity as a predictor of clinical outcome, see Horn et al. (2017b). In this cohort patients qualified for DBS if they had been diagnosed with PD for a duration more than 4 years, were older than 45, had greater than 30% reduction in Unified Parkinson’s Disease Rating Scale Part III (UPDRS) score for the levodopa challenge, and neuropsychological testing excluded DBS contraindications, such as a score of less than 130 on the Mattis Dementia Rating Scale, major depression, or acute psychosis. Bilateral STN surgery included use of microelectrode recordings to determine electrode placement of quadripolar electrodes (model 3389, Medtronic, Minneapolis, MN, United States). All patients had preoperative magnetic resonance imaging (MRI), postoperative computed tomography (CT), long-term clinical follow-up to optimize stimulation settings, 1-year UPDRS III data and levodopa equivalent daily dose (LEDD). Please see Horn et al. (2017b) for further details.

Two patients from this cohort were disqualified from this study: one did not have a complete UPDRS III exam and another patient’s targeting images had an insufficient number of MRI slices for our analyses.

### Study design

This was a retrospective, single-center evaluation of the relationship of lead location and UPDRS III outcome using data collected from patients who had previously undergone bilateral STN DBS at Wurzburg, Germany. The objective of the study was to assess whether the stimulation site determined from the MRI-defined STN using high precision imaging technology, SIS System (Surgical Information Sciences, Plymouth, MN, United States), to visualize the individual patients’ STN, predicted the patient’s motor outcome. The location (anterior to posterior and medial to lateral location) of each active contact (cathode) was determined and then correlated to the change in motor outcome 1 year following DBS surgery.

Motor outcomes were previously evaluated using the UPDRS III with a total possible score of 132. Within this exam, 88 possible points were allotted to extremity motor scores (Questions #3 rigidity upper/lower extremities, #4 finger tapping, #5 hand movements, #6 pronation/supination of hands, #7 toe tapping, #8 leg agility, #15 postural tremor of hands, #16 kinetic tremor of hands, and #17 rest tremor amplitude upper/lower extremities). These questions were used to develop a “hemi-body UPDRS” motor score (44 possible points/side) that was correlated to contralateral STN stimulation since electrode locations were evaluated independently for each side. Preoperatively motor scores were recorded off and on medication after overnight withdrawal from antiparkinsonian medication for greater than 12 hours. At the 1-year follow-up visit, UPDRS motor scores were collected in the medication off/stimulation on condition, and the optimized stimulation settings of activated contact(s), amplitude and frequency were reported.

### Subthalamic nucleus visualization and image analysis

**FIGURE 1:**
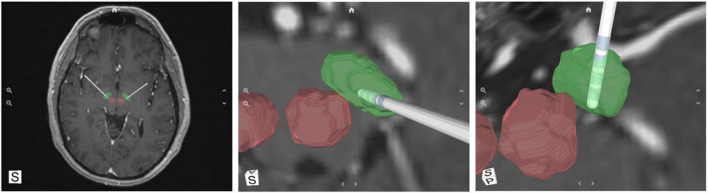
A representative example of the SIS System output; a 3D reconstruction of subthalamic nucleus (STN) (green), red nuclei (red) and the deep brain stimulation (DBS) electrode with its corresponding individual contacts.

Retrospective standard preoperative MRI and postoperative CT images were uploaded to a web-based portal and analyzed with the SIS System (Duchin et al., 2018; Shamir et al., 2018; Kim et al., 2019). The system incorporates a machine-learning algorithm that was trained on a database of 7T MR images with manually defined labeled data, to augment anatomical structures of the brain from standard imaging. The system is validated to visualize patient-specific location of the STN, its borders, and lead location within less than one millimeter of ground truth (Duchin et al., 2018; Shamir et al., 2018; Kim et al., 2019). This differs from other available visualization methods in that it recognizes varying anatomical shapes, sizes, and orientations of each patient rather than utilizing atlas coordinates. This software is intended for visualization of anatomical structures for planning of DBS and for post-operative reconstruction of lead/contact locations, the latter of which was the purpose of this evaluation. The SIS System analyzed pre-operative 1.5T or 3T MR and postoperative CT images and rendered a 3D model of each patient’s STN and visualization of the electrode contacts (Figure 1).

**FIGURE 2:**
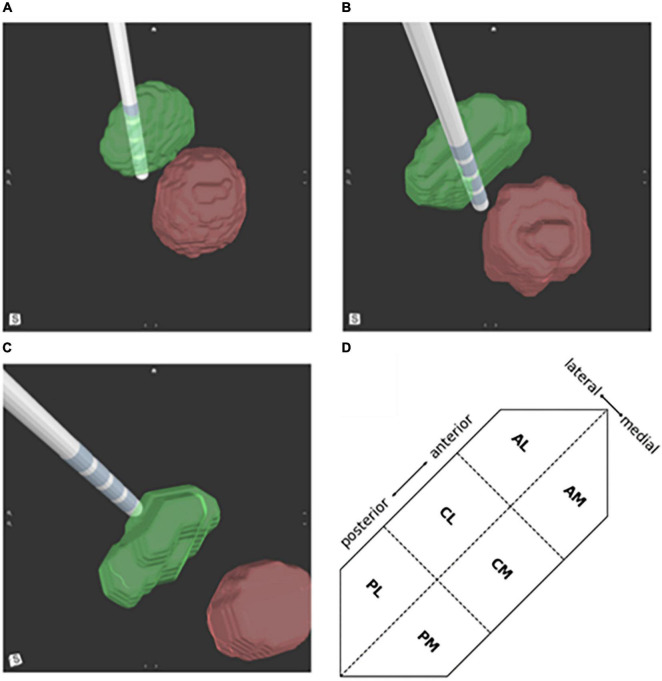
Deep brain stimulation (DBS) lead location in respect to the subthalamic nucleus (STN) (green) and red nucleus (red). Activated lead contacts were evaluated for their position as **(A)** fully within the STN border, **(B)** partially inside the STN border, or **(C)** outside the STN. **(D)** A schematic illustration of a left STN showing division of the 6 regions; PL, posterolateral; PM, posteromedial; CL, centrolateral; CM, centromedial; AL, anterolateral; AM, anteromedial.

The 3D images and reported optimal, activated contact(s) were evaluated by a designated assessor who was blinded to patient outcomes. The assessor reported the location of the activated contact(s) compared to the proximity to the STN border. The location was first described as fully inside the STN, partially inside the STN if the active contact overlapped the border of the STN, or outside the STN if the active contact was fully outside the border of the STN (Figures 2A–C). Then the assessor reported the location of each activated contact(s) as belonging to one of the predefined six segments of the STN based on their anterior-posterior and medial-lateral location: anterolateral (AL), anteromedial (AM), centrolateral (CM), centromedial (CL), posterolateral (PL), and posteromedial (PM) (Figure 2D). Since retrospective preoperative imaging was used parcellation of the STNs to their functional motor, associative and limbic territories was not available.

### Statistical analysis

Data were summarized using descriptive statistics for continuous variables. Analysis of covariance (ANCOVA) were used to test for differences in follow-up stimulation of UPDRS scores after adjusting for differences in baseline UPDRS scores (*p*-value ≤0.05 is considered significant). Categorical variables were described using count, percentage, and sample size.

The relationship of the active contact location on the mean percentage change in the hemi-body UPDRS III score from baseline to the 1-year follow-up measurement was calculated from the preoperative medication off condition to follow-up medication off/stimulation on condition.

## Results

### Demographics

Forty-two patients qualified for this retrospective study, however two were excluded from analyses because a 3D model was unable to be generated due to significant brain shift and a code issue that was resolved in a later version of the SIS System. Therefore 40 patients and 80 DBS electrode/active contact locations were included in this study. The mean age at the time of DBS surgery was 60.8 years (SD ± 7.8, range 46.5–74.8) and 70% were male. The mean duration of PD in this group was 12.3 years (SD ± 4.2, range 5–21).

Preoperatively, patients were taking an average LEDD of 1477 mg (SD ± 599, range 290–2,850). Baseline UPDRS III score off medication was 50.1 (SD ± 12.7, range 26–78), with medication it was 18.6 (SD ± 10.3, range 2–46), representing a mean improvement in motor function of 62.9%.

### Relationship of electrode contact location to deep brain stimulation motor outcomes

Fifty-six percent (45/80) of the active electrode contacts were fully within the borders of the STN, while 20% (16/80) were partially within the STN and 24% (19/80) were completely outside the STN border (Table 1).

**TABLE 1:** Performance of stimulated active contacts based on implanted location relative to the subthalamic nucleus (STN).

Active contact location (N)	Baseline body side UPDRS III 0–88 (Meds OFF) mean ± SD	Body side UPDRS III with Stimulation 0–88 (Meds OFF) mean ± SD	Body side change from baseline mean ± SD	*p*-value compared to fully inside	Body side motor improvement	LEDD reduction mean (mg)	LEDD reduction	Current amplitude mA mean	Stimulation frequency Hz mean
Fully inside the STN (45)	16.3 ± 5.2	6.9 ± 4.0	−9.4 ± 6.3	n/a	57.7%	−994	68.2%	3.0	134.7
Partially inside the STN (16)	14.6 ± 5.0	7.4 ± 3.3	−7.3 ± 5.2	0.586	49.6%	−611	44.2%	3.2	151.3
Outside of the STN (19)	15.3 ± 6.0	8.4 ± 4.4	−6.9 ± 6.9	0.142	45.0%	−845	52.6%	3.7	151.1
**STN segment: fully and partially inside the STN (N)**				***p*-value Compared to PL STN**					
Anteromedial (1)	11.0 ± 5.4	7.0 ± 4.1	−4.0 ± 5.6	0.216	36.4%	−742	62.9%	3.9	165.0
Anterolateral (3)									
Centromedial (16)	16.4 ± 5.2	7.9 ± 4.2	−8.5 ± 5.3	0.039	51.9%	−881	56.7%	3.0	143.1
Centrolateral (24)	15.7 ± 5.4	7.5 ± 3.8	−8.2 ± 6.2	0.048	52.4%	−885	65.3%	2.9	139.8
Posteromedial (6)	16.8 ± 4.4	7.2 ± 4.1	−9.7 ± 5.5	0.222	57.4%	−372	30.5%	3.3	141.7
Posterolateral (11)	16.9 ± 5.0	4.8 ± 2.6	−12.1 ± 6.3	n/a	71.5%	−1271	76.6%	3.1	120.5

ANCOVA of stimulation scores adjusted for baseline. Comparison of hemi-body Unified Parkinson’s Disease Rating Scale (UPDRS) scores before and after deep brain stimulation (DBS) surgery, levodopa dosage (mg) and stimulation settings (mA, Hz) for different regions of the STN.

Improvement of hemi-body UPDRS III scores from baseline to 1 year were compared to the contralateral STN active contact location. Motor function improved in 90% (72/80) of implanted DBS locations. The greatest motor improvement occurred when the active contacts were located fully within the borders of the STN (57.7%, −9.4 points). Improvement was less for contacts located partially within the STN (49.6%, −7.3 points) and those outside the STN demonstrated the smallest improvement (45.0%, −6.9 points) in motor function (Table 1). Patients with active contacts entirely within the STN border showed a greater reduction in LEDD, lower voltages and stimulation frequencies relative to contacts outside or partially in the STN (Table 1). These differences were not statistically significant.

A 2nd level analysis was performed to correlate clinical outcomes and lead location relative to the anterior to posterior and medial-lateral locations of the active contacts. Six segments were defined based on the relative location of the active contact (see section “Materials and methods” and Figure 2D). Of the active contacts that were partially or fully within the STN, 39% and 26% were in the CL and CM regions of the STN, respectively. The remaining active contacts were in the following segments of the STN: PL 18%, PM 10%, AL 5%, and AM 2% (Table 1). The greatest improvement in motor function was found with active contacts located within the PL segment of the STN. These contacts (*n* = 11) demonstrated 71.5% (−12.1 ± 6.3) hemi-body improvement compared to pre-DBS hemi-body motor scores. The second greatest motor improvement was reported for active contacts (*n* = 6) located within the PM area of the STN with 57.4% improvement (−9.7 ± 5.5). The majority of the active contacts were located in the CL (*n* = 24) and CM (*n* = 16) segments of the STN; these contacts demonstrated a mean change of 52.4% (−8.2 ± 6.2) and 51.9% (−8.5 ± 5.3) improvement in motor function, respectively. The AL and AM locations of the STN had the fewest number of leads (AL = 3, AM = 1) and showed the least amount of clinical improvement. Combining the four leads the average improvement was 36.4% (−4.0 ± 5.6). The PL segment demonstrated a statistically significant difference in clinical benefit compared to the CL (*p* < 0.05) and CM (*p* < 0.04) segments (Table 1).

Further evaluation of electrodes near the PL segment demonstrated that motor function improved by 71.4% (−12.0 points) for active contacts fully within the STN, 72.2% (−13.1 points) for active contacts partially within the PL STN, and 53.0% (−8.8 points) for active contacts just outside the PL STN.

**FIGURE 3:**
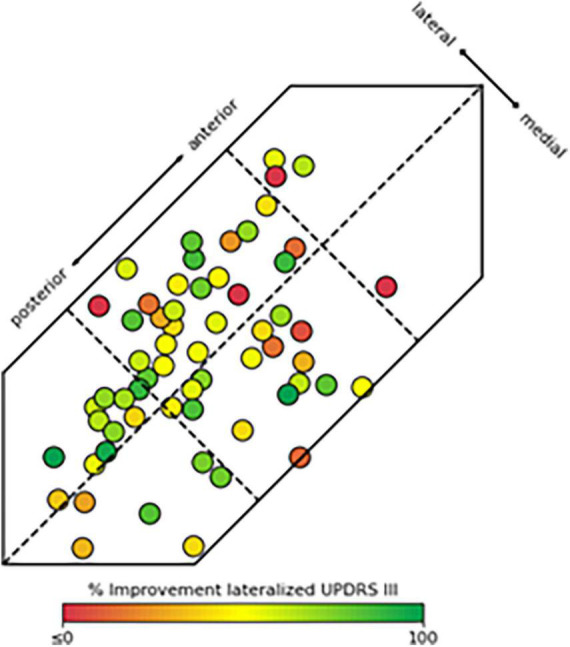
Location of active contacts fully or partially within the subthalamic nucleus (STN) and their corresponding hemi-body motor improvement Unified Parkinson’s Disease Rating Scale (UPDRS% improvement). These data are represented as a heat map with lower or negative UPDRS improvement shown in red (≤0%) to greatest UPDRS improvement shown in bright green (100%). Contacts partially within the STN may appear fully within the STN in the figure as their locations were dorsal or ventral to the body of the STN region in the 3D model.

When examining the consistency of benefit in clinical outcomes in relation to their location within different segments of the STN, i.e., posterior, central, and anterior lead placements, the likelihood of obtaining clinical improvement greater than or equal to 50% was significantly greater with leads placed in PL compared to CL or CM (*p* < 0.05, Table 2 and Figure 3). Similarly, there was an increasing likelihood of obtaining benefits less than 40–50% with active contacts that were anterior to PL, i.e., within CM or CL. There were not enough data points to compare these data to AL or AM locations, however the variability in response with lead placement in more anterior locations was greater, and improvement in motor function was less compared to those placed posteriorly in the PL site (Table 2). Figure 3 provides a pictorial view showing the active contact location to clinical outcome represented by a heat map for clinical outcomes within the six regions of the STN.

**TABLE 2:** Percentage of active stimulated contacts at increasing motor improvement defined in each subthalamic nucleus (STN) segment when contacts are located fully or partially inside the STN borders.

STN segment (N)	Active contacts with <40% improvement (%)	Active Contacts with <50% improvement (%)	Active contacts with ≥50% improvement (%)	Active contacts with ≥60% improvement (%)	Active contacts with ≥70% improvement (%)
AM + AL (4)	50	50	50	0	0
CM (16)	25	44	56	38	25
CL (24)	25	42	58	38	29
PM (6)	33	50	50	50	50
PL (11)	18	18	82	73	45

### Levodopa equivalent daily dose and stimulation settings

For all patients pre-operative mean LEDD was 1,477 mg (±599). One year following DBS surgery this was reduced by 59.7% to 595 mg (±408). The mean LEDD reduction for active contacts in the PL STN was 76.6% (−1271 mg) at 1 year. This finding was statistically significant when compared to the overall LEDD reduction for patients with active contacts in the other segments (58.4%, −811 mg, *p* = 0.034). Mean LEDD reduction for active contacts in the other segments was 30.5% (−372 mg) in PM, 65.3% (−885 mg) in CL, 56.7% (−881 mg) in CM, and 62.9% (−742 mg) in combined anterior segments (Table 1).

Given that patient response to Levodopa is considered a predictor of the potential benefit that can be obtained with DBS (Defer et al., 1999; Welter et al., 2002; Lang et al., 2006; Machado et al., 2012) we also examined the relative effect of DBS to that obtained with medical therapy. PL STN stimulation provided motor benefit that was comparable to or greater than that provided by Levodopa alone. Subjects with leads in the PL STN had an overall UPDRS III improvement of 65.3% with medication, compared to 71.5% improvement with DBS. The PL segment of the STN was the only segment to demonstrate greater motor improvement with stimulation alone than with medication alone. Electrodes in the PL showed 109.4% of the medication response on the hemi-body motor score, followed by 94.2% in CM, 80.8% in PM, 78.9% in CL, and 48.2% in the anterior segments.

Mean stimulation settings in the 40 patients were 3.1 mA (±0.9) and 140.9 Hz (±28.9) at the 1-year follow-up visit. The lowest mean frequency occurred when active contacts were located fully within the STN and current amplitudes were lowest for active contacts located fully or partially within vs. those outside the STN (*p* < 0.05). Average stimulation parameters were 3.0 mA and 134.7 Hz for active contacts fully within the STN, compared to 3.2 mA and 151.3 Hz for contacts partially inside the STN border and 3.7 mA and 151.1 Hz for active contacts outside the border of the STN (Table 1).

## Discussion

### The posterolateral subthalamic nucleus and deep brain stimulation

This study demonstrated that the location of the stimulating DBS contact within the STN can have a substantial effect on the outcome of patients undergoing DBS procedures for PD. While 93% of patients in this study experienced a net improvement of 52.9% in motor function with STN DBS, there were clear differences based on the location of the active contact as well as whether it was fully within, partially within, or outside the STN border. There was significantly more benefit when stimulation contacts were within the PL region compared to CM and CL. There was also less variability in clinical outcomes for leads placed in PL compared to those in CM, CL or more anterior locations.

Reduction in antiparkinsonian medication with active contacts located within the PL region was also greater than what was observed in the other segments, and patients with PL DBS showed motor improvement comparable to or greater than that experienced with medication alone. Indeed, the overall DBS effect in the PL segment of the STN was 109.4% of the medication response (*p* > 0.05). While the small numbers of contacts located in this region precluded any conclusions over the relative significance to other segments, the vast majority of studies have rarely reported DBS improvements that were greater than what has been reported with medication alone (Deuschl et al., 2006; Follott et al., 2010; Okun et al., 2012; Vitek et al., 2020). One potential explanation for this observation could be its location relative to nigrostriatal dopaminergic fiber pathways, another could be differences in phenotype between patients implanted in PL vs. other segments or the fact we assessed hemi-body improvement and excluded axial motor signs given the study design. Answers to these questions will require further study in a larger cohort of patients.

In addition to producing greater clinical benefit and larger reductions in antiparkinsonian medication, less stimulation current was required to produce these changes with active contacts in the PL region. Mean stimulation settings in the PL STN indicated a modest current requirement that was generally lower than most of the settings for electrodes in the other five segments.

The greater benefit observed in the PL region is consistent with previous reports that optimal motor outcomes occur when leads are placed in the sensorimotor (SM) region, which lies in the dorsal portion of the posterolateral STN (Richardson et al., 2009; Wodarg et al., 2012; Guo et al., 2013; Schrock et al., 2021; Zhang et al., 2021). This is also the region where beta activity is at its greatest (Zaidel et al., 2010; de Solages et al., 2011; Wodarg et al., 2012; Fernández-García et al., 2017; Horn et al., 2017b; Tinkhauser et al., 2018; Aman et al., 2020). While there is agreement by many that there is an optimal location for placement of the DBS lead, there remains controversy over the actual location that provides the greatest motor benefit (Wodarg et al., 2012; Bot et al., 2018). Garcia-Garcia and Guridi (2016) created an adaptable 3-D atlas and reported that the rostral and most lateral parts of the STN provide the greatest benefit. Others have reported that outcomes are comparable regardless of the location of the lead within the STN (McClelland et al., 2009; Welter et al., 2014). There are a number of factors that likely contribute to the differing results reported across studies regarding the relative importance of lead location to motor benefit. The use of atlas-based reconstructions does not account for the anatomic variability in size, shape and geometric configuration of the STN that is present across patients (Duchin et al., 2018; Plantinga et al., 2018). Such a one-size-fits-all approach can easily lead to errors in the accuracy of targeting and post-surgery reconstructions of the locations of contacts within the target. In addition to anatomic variability is the fact that the functional subregions of the STN, motor, associative and limbic regions, also differ in their relative distribution (Plantinga et al., 2018). While a general organizational pattern has emerged for these functional subregions with a posterolateral motor territory overlapping with a central associative region and limbic territory in the anteromedial portion of the STN, the volume occupied by each region and degree of overlap between functional zones can vary from patient to patient (Plantinga et al., 2018).

Another factor that could play a significant role in determining outcomes is the difference in functional connectivity within subcortical-cortical circuitry that exists across PD patients. Work by Horn et al. (2017b) reported that both structural and functional connectivity were independent predictors of clinical improvement. More recent studies have also hypothesized that the orientation of the current field relative to the target and axonal projections from, to, and adjacent to the target may influence the relative degree of activation of these axonal pathways (Slopsema et al., 2018). While there are a number of variables that likely play a role in determining the degree of clinical benefit, the present data along with previous studies provide compelling evidence in support of targeting the “sensorimotor” region, mainly the PL section of the STN.

### Improvement in non-motor regions

While contacts stimulating the posterolateral regions of the STN gave the greatest and most consistent benefit in this retrospective study we found that more centrally located regions were also associated with improvement in motor signs, albeit less consistently so. Without parcellation studies we were not able to determine the location of the lead relative to motor, limbic or associative territories. It is possible some of the more posteriorly active contact locations in CM and CL could have included sensorimotor regions. It is also possible that changes in premotor/prefrontal cortical regions during stimulation in the associative territory could also account for this finding (Rowe et al., 2002; Schroeder et al., 2003; Wang et al., 2017). In addition, current spread posteriorly into the adjacent SM region, activation of fiber pathways leaving the SM region and coursing through associative regions of the STN, as well as activation of adjacent cerebellothalamic, nigrostriatal or pallido-thalamic pathways could also play a role.

### Role of patient-specific postoperative lead reconstructions within the subthalamic nucleus

This is the first study using the SIS software to automatically and without user intervention identify the DBS lead and contact locations with respect to the defined borders of the STN based on direct visualization of the patient’s standard clinical brain imaging (Duchin et al., 2018; Shamir et al., 2018; Kim et al., 2019). This software differs from other systems that provide 3D modeling in that SIS is patient-specific with greater accuracy than its atlas-based counterparts (Pallavaram et al., 2015; Duchin et al., 2018; Shamir et al., 2018; Kim et al., 2019). Unique to this study was the ability to utilize this technology to precisely determine the location of active DBS contacts within each patient’s STN and the relationship of those contacts to clinical motor improvement.

Atlas-based targeting has been used for decades yet there remains inaccuracy due to anatomical subject variabilities and registrations issues. The ability to develop patient-specific targeting will be important if we are to consistently provide the best motor outcomes for each patient. It will depend on visualization of each patients target structure for accurate lead implantation and the ability to determine the precise location of the implanted DBS lead and contacts with respect to the anatomical borders of the STN. Novel approaches, such as the one presented here, can address visualization constraints and play a vital role in the future to allow individualized reconstructions that enable one to develop automated programming algorithms (Guo et al., 2007; Pallavaram et al., 2015; Plantinga et al., 2018; Kim et al., 2019; Solomon et al., 2021).

### Limitations

Hemi-body UPDRS was used to draw conclusions about implanted electrode location. This approach while helpful to assess the relative effect of stimulation on one side vs. the other only uses a possible 88 of 132 points on the UPDRS III scale to differentiate left and right-side motor function. This assessment does not account for other motor functions like gait, speech, posture, etc., that cannot be attributed to just one electrode location on one side. Since the hemi-body UPDRS scores are only a subset of the entire exam, the scores are likely to show greater improvement given motor signs that are generally less responsive to DBS are not included. It was also difficult to draw statistical significance with the current sample size given the majority of contacts were placed in more central regions of the STN, limiting our ability to perform multiple comparisons across regions.

Another limitation to this study was the use of DBS cases that had been implanted at another center which limited the amount and type of data available from patient records. The lack of side effects reported in the data set and minimal neuropsychological data that limited our ability to assess the relative effect of DBS in more anterior non-motor sites that can limit overall benefit. Since these patients were implanted prior to availability of directional/segmented leads, it is unclear what effect current steering could have on improving motor function in patients whose leads may have been placed more anteriorly or partially inside but adjacent to the PL region. Also absent from analysis was the volume of tissue activation which could have provided additional correlation with lead locations.

Last, we did not assess the relative effect of dorsal vs. ventral locations in the different regions of the STN. While the majority of reports studying dorsal vs. ventral lead location have suggested dorsolateral portions of the STN are superior to more ventral locations we did not include this comparison and focused on the anterior-to-posterior and medial-lateral segments of the target.

## Conclusion

This study provides further support for the hypothesis that DBS lead location plays a significant role in determining clinic benefit. It also demonstrated that patient-specific visualization of the anatomical target with postoperative localization of individual contacts can offer insight into programming strategies and allow physicians to develop patient-specific targeting algorithms based on accurate visualization of each patients STN. This is particularly important given the variation in size, shape, and geometric configuration of the STN across patients. Although evidence was provided in support of the role of lead location and clinical benefit and proposes the PL region of the STN as the preferred site for STN DBS, the current sample size does not allow for definitive statistical significance in all cases and did not address the relative effect of different contact locations on individual motor signs, i.e., patient phenotype. More work will be required to validate these findings and determine the relative role of active contact location, not only in overall motor improvement but in individual motor signs as well as cognitive function if we are to develop patient-specific targeting of PD patients undergoing STN DBS. A similar approach will be necessary as we explore additional targets for other disorders.

## Data availability statement

The data generated during this study are available from the corresponding author on reasonable request.

## Ethics statement

The studies involving human participants were reviewed and approved by Ethics Committee of the University of Würzburg. The patients/participants provided their written informed consent to participate in this study.

## Author contributions

JLV contributed to conceptualization, methodology, investigation, formal analysis, writing, and supervision. RP contributed to formal analysis, investigation, and writing—review and editing. LI contributed to formal analysis, data curation, writing, and visualization. MR and JV contributed to methodology, investigation, resources, and writing—review and editing. NH contributed to conceptualization, software, formal analysis, writing, and visualization. All authors contributed to the article and approved the submitted version.
